# Being honest won’t pay. Seven- but not 5-year-olds begin to predict that others will lie for reputational reasons

**DOI:** 10.1371/journal.pone.0317334

**Published:** 2025-01-10

**Authors:** Mareike Klafka, Ulf Liszkowski

**Affiliations:** Department of Developmental Psychology, University of Hamburg, Hamburg, Germany; NTT Communication Science Laboratories, JAPAN

## Abstract

Children begin to manage their reputation around school-age, but it remains unclear when they start to explicitly reason about reputational strategies such as lying from a third-person perspective. The current study investigated whether 5- and 7-year-old children would explicitly predict reputational lying in the context of a third party interaction. Participants were told hypothetical stories and asked to predict whether a protagonist would lie to a peer character about a selfish resource allocation. Results revealed that about half of the 7-year-olds and neglectable few of the 5-year-olds began to predict that the protagonist would lie to his peer out of reputational concern and whitewash the selfishly distributed amount. The prediction of reputational lying did not differ for ingroup or outgroup third parties. Seven-year-olds justified their prediction of a lie with reference to how the protagonist would look to others. While reputational lying has been shown in 5-year-olds in comparable interactive scenarios with peers, a more abstract, explicit understanding of reputational lying seems to be a more complex cognitive ability, emerging around the age of 7 years.

## Introduction

Social and moral evaluations are important competencies in the process of cooperation and partner choice [1]. Humans are eager to demonstrate that they are a good partner for collaboration and, in contrast to other great apes, “simulate the perspective and evaluations of others for the purpose of actively managing the impression they are making on others” ([1], p.281). Social evaluation concerns emerge in the preschool years, when children begin to modify their behavior in the presence of others [2, 3] or in the knowledge that they are being assessed by others even when they are alone [4, 5]. A recent study suggests that around the age of 5 years, children begin to lie about their resource allocations in order to manage their reputation [6]. In the study, participants played a mini dictator game and could share some of their stickers with another (anonymous) child. Children consistently distributed the stickers in their favor. However, when subsequently asked by a peer (an ingroup or an outgroup member) how many stickers they had shared, they exaggerated the number of stickers. Thus, children seem to use lying as a legitimate strategy to manage their reputation. At the same age children are less likely to cheat, if they are told that they have a positive reputation to maintain [7]. Thus, in their direct interaction with others, children engage in strategic behaviors to manage the impressions others form of them, including lying and cheating.

The current study is concerned with the emergence of explicit knowledge about reputation management through strategic lying from a hypothetical third-person perspective. Interactional behavior is often taken to reflect practical knowledge in second-person engagement [8]. In contrast, explicit, theory-like organized knowledge extends to predictions and justifications of others’ behaviors from a practically rather uninvolved third-person perspective [9]. For example, in the area of Theory-of-Mind research, early implicit skills are typically contrasted with later explicit knowledge about others’ minds [10, 11]. Investigating the emergence of explicit forms of knowledge about reputation managing strategies will provide further insights into the ontogenetic and cultural development of social and moral evaluations and reputational cognition. When will children begin to predict, in the context of a third-person hypothetical scenario, that someone will lie to manipulate another person’s belief about her?

Findings suggest that around the age of 8 years, children start to reason about their own reputation [2] and draw inferences about reputational motives [12]. For instance, children were presented hypothetical vignettes of a protagonist committing a moral or social-conventional transgression [13]. They were asked to actively immerse themselves in the scene and to state how they would feel and what they would do if they were the protagonist. With age, children became more likely to refer to reputational concerns about what others would think about them as justifications for their chosen responses, especially in the context of social-conventional transgressions. In another study [14], 8- and 9-year-olds inferred from a vignette that children who had offered gifts to others in public compared to private settings might have an ulterior motive to enhance their reputation, whereas 6- and 7-year-olds did not make this inference. While all children were able to reason about others’ mental states, only older children identified reputational motives to explain others’ behaviors.

A recent study suggests that around the age of 6–7 years, and in some contexts around the age of 5 years, children expect different kinds of behaviors depending on whether an agent has reputational versus intrinsic motives (e.g. whether the agent wants to “appear” versus “be” smart) [15]. In the study, children were asked in a forced-choice question which character they thought would lie to cover up their poor performance, and which character they thought would lie about their success to protect someone’s feelings. In line with the expectations, children predicted the reputationally motivated character to be more likely to lie in order to cover up their poor performance and to be less likely to lie about their success to protect someone else’s feelings. In a further study [16], 6-11-year old children were asked to reason about the accuracy of individuals’ statements about themselves (“Do you think they might tell me that they do a lot of nice things for other people even if they know that’s not true?”). Results suggest that with age children become more likely to assume that others might present false claims about themselves. However, results were influenced by cultural differences as a reverse pattern of results was found for children from China compared to children from the United States. These cultural differences might be due to modesty norms in China, which prescribe the concealment of one’s positive characteristics.

As reputation management enhances partner choice for cooperation, several studies have investigated how children’s reputation management is related to their later partner choice and how it is influenced by specific characteristics of the potential partner. From age 5, children selectively choose partners for cooperation depending on their task-relevant qualities, whereas younger children choose their partners indiscriminately [17]. Further, 5-year-olds share more resources with a partner who they know will later have the opportunity to reciprocate [18]. In the current study, from the perspective of explicit knowledge, we therefore also investigated whether children’s explicit understanding of third-party reputation management includes predictions about others’ partner choices.

Furthermore, reputation management has been shown to be stronger for ingroup than outgroup members [19]. Children are more willing to tell a prosocial lie if they receive an undesirable gift from an ingroup compared to an outgroup peer [20]. At the age of 11 years, they also rate prosocial lies told to an ingroup member more positively than those told to an outgroup member [20]. This ingroup-bias likely rests on (indirect) reciprocity, as children reliably identify individuals who might subsequently be in a position to help them, and they strategically invest in their relations and reputations with those individuals [19]. Therefore, in the current study, from the perspective of explicit knowledge, a further question is whether the third-party characters’ group memberships (whether they are from the same group or different groups) will affect children’s predictions of lying and their reputational justifications.

We adopted the general structure of a previous study which had investigated children’s lying in their direct interactions as a reputational strategy [6]. In analogy, we created hypothetical stories of third-party interactions, in which characters engaged in resource allocations (mini-dictator game). Participants were asked to predict a protagonist’s behavior who interacted with a peer after a selfish resource allocation. They were told that a protagonist received 10 cookies and could decide whether to share some of the cookies with another (hypothetical) child. The protagonist distributed the cookies in his favor and only shared one cookie with the hypothetical child in the story. Then, a peer character who previously had distributed her cookies fair, asked the protagonist how many cookies he had donated to the other child. We manipulated whether the peer character would belong to the same group and have the same gender as the protagonist (ingroup condition) or whether the peer character would belong to a different group and have a different gender than the protagonist (outgroup condition). Participants were then asked to predict the number of cookies the protagonist would state he had donated when asked by the peer character, and to provide a justification for their prediction. In addition, they were asked whether the peer character would later choose the protagonist to play with. We tested 7-year-old children to be able to capture the beginnings of an explicit understanding of reputational lying [see 15]. We further tested 5-year-olds who show reputational lies themselves [6], to test whether their skills already involve a theory-like explicit understanding of reputational lying, independent of their own situation and activities, instead of a rather practical use of reputational lying. This is analogous to findings on false belief understanding suggesting that 3-year-olds use a practical false belief understanding to guide their intentional actions, but do not possess an explicit understanding of false belief, which emerges 1–2 years later [10, 11].

If children have an explicit understanding of reputational around the same age they show this strategy themselves in interactions with peers, 5-year-olds should predict that the protagonist who distributed a resource selfishly would lie to the peer character in order to manage his reputation. If the explicit understanding of reputational lying is a more complex cognitive ability, only 7-year-olds should predict reputational lying and justify their prediction with reference to reputational concerns. If children understand group biases in the context of reputation management, in line with previous research they should predict more reputational lying towards an ingroup than towards an outgroup member [19, 20]. If reputational concerns are the very reason why children expect the protagonist to tell a lie, they should justify their response with reference to how the protagonist would look to others and with reference to situational outcomes (e.g., “So they’ll be friends”) more often than children who predict an honest response. Accordingly, children who predict an honest response should either justify their prediction more often with reference to social or moral standards or with motives that do not clearly involve reputational concerns.

We explored two further aspects relevant to an explicit conceptual understanding of social and moral evaluations. First, research with adults suggests that even prosocial lies told with the intention to benefit another person harm integrity-based trust, that is, the willingness to rely on the veracity of another person, e.g. on her word or promise [21]. This effect is independent of whether participants directly experience deception or observe deception in a third-person scenario. Since reputational lies question a person’s veracity, they might harm integrity-based trust in a similar way as prosocial lies. In the current study, we therefore explored whether children who predict reputational lies will evaluate the protagonist as less trustworthy and expect him to keep a promise less often than children who predict honesty. Previous studies have shown that around the age of 5 years, children begin to explicitly reason about the reliability of others [22] and to include recent track record of accurate or inaccurate information [23] and to consider motivations of others when evaluating their credibility [24]. The current study will thus expand prior findings on the relation between lies and trustworthiness with children.

Second, reputational lies may affect children’s willingness to subsequently cooperate with the lie-teller. On the one hand, honesty and trust are crucial to cooperation and a lie-teller might lose his opportunity to be part of the group. On the other hand, reputational lies prove the lie-teller to be able to internalize the group’s norms and to present himself in a way fitting into the group. Thus, the lie-teller might still be a good partner for cooperation. The current study explored children’s willingness to be friends with the protagonist (see [25]) after they had predicted him to either lie or be honest.

## Materials and methods

### Participants

In total, 64 monolingual German-speaking children were tested in this study. An a priori power analysis using G*Power 3 for a t-test between the mean predicted answer and the actual donation of one cookie based on α = 0.05, a target level of statistical power of .80 and effects of Cohen’s *d* = .44 (based on [7]), suggested a sample size of *n* = 34. The final sample consisted of 32 5-year-olds (16 girls; mean age = 5 years and 7 months, SD = 3.65 months) and 32 7-year-olds (16 girls; mean age = 7 years and 8 months, SD = 2 months). Participants were recruited from April, 7th, 2021 to November, 27th, 2021 from the department’s database of children whose parents had previously agreed to be contacted for study participation. General exclusion criteria were visual impairments or hearing loss as well as the interruption of the study due to technical problems or obvious distraction of the participant. No child had to be excluded because of these criteria.

### Design & procedure

The study was noninvasive and conducted in accordance with the Declaration of Helsinki. The procedure was approved by the local ethics committee of the Faculty of Psychology and Human Movement Science at University of Hamburg. The study was conducted online via Zoom. Children were tested individually in a within-subjects design with two conditions: the ingroup condition and the outgroup condition. In an e-mail sent to the parents prior to the study, they were instructed to create appropriate testing surroundings without distractions. Before the beginning of the experiment, parents gave written informed consent and the child gave verbal informed consent. Children were told that they were to hear a series of stories and would then be asked a few questions to hear what they think about it. The stories were presented as series of cartoon-style drawings in a slide show. The text was read out by the experimenter. The stories are provided in the S1 File.

#### Practice story

After a short warm-up with the experimenter, the study started with a practice story to familiarize the child with the general procedure of the study and to make sure the child would listen to the experimenter and remember all relevant information. In the practice story, the experimenter presented a picture of a lion, a duck and a chocolate and stated that the chocolate would belong to the lion. Then the pictures disappeared and the experimenter asked the child to remember to whom the chocolate belonged. If the child answered incorrectly, the experimenter repeated the instruction. If the child answered correctly, the experimenter continued with the procedure.

#### Test stories

Next, children were presented two test stories. The order of the two stories was counterbalanced across children. In the ingroup condition, the experimenter presented a picture of two children matched to the gender of the participant, e.g. two boys (Tom and Max) belonging to the same group (e.g. the “star group”) and collaborating on a joint activity (e.g. building a tree house). Next, the experimenter presented a picture of one of the boys (Tom) and told the participant that Tom received ten cookies, which he really liked. The participant was asked whether he also liked cookies. Next, the experimenter told the participant that Tom could decide whether he wanted to keep all cookies for himself or donate some of the cookies to another child of his group. The experimenter continued that Tom had decided to give one cookie away and to keep nine cookies for himself, because he wanted to eat nine of them. Next, the experimenter asked the participant two control questions (“To what group does Tom belong?” and “How many cookies did Tom donate to the other child?”) to check whether the participant would remember the relevant information. If the participating child did not remember the number of donated cookies correctly, he was asked to remember how many cookies Tom had kept for himself. If children gave an incorrect answer, they were excluded from analyses. If they gave a correct answer, the experimenter repeated their response.

Next, the experimenter presented a picture of the other boy (Max) and told the participant that Max received ten cookies as well. The experimenter told the participant that Max could decide whether he wanted to keep all cookies for himself or donate some of the cookies to another child of his group. The experimenter continued that Max had decided to give five cookies away and to keep five cookies for himself, because he considered this fair. Again, the experimenter asked the participant the two control questions, repeating his response, if he gave the correct answer.

**Fig 1:**
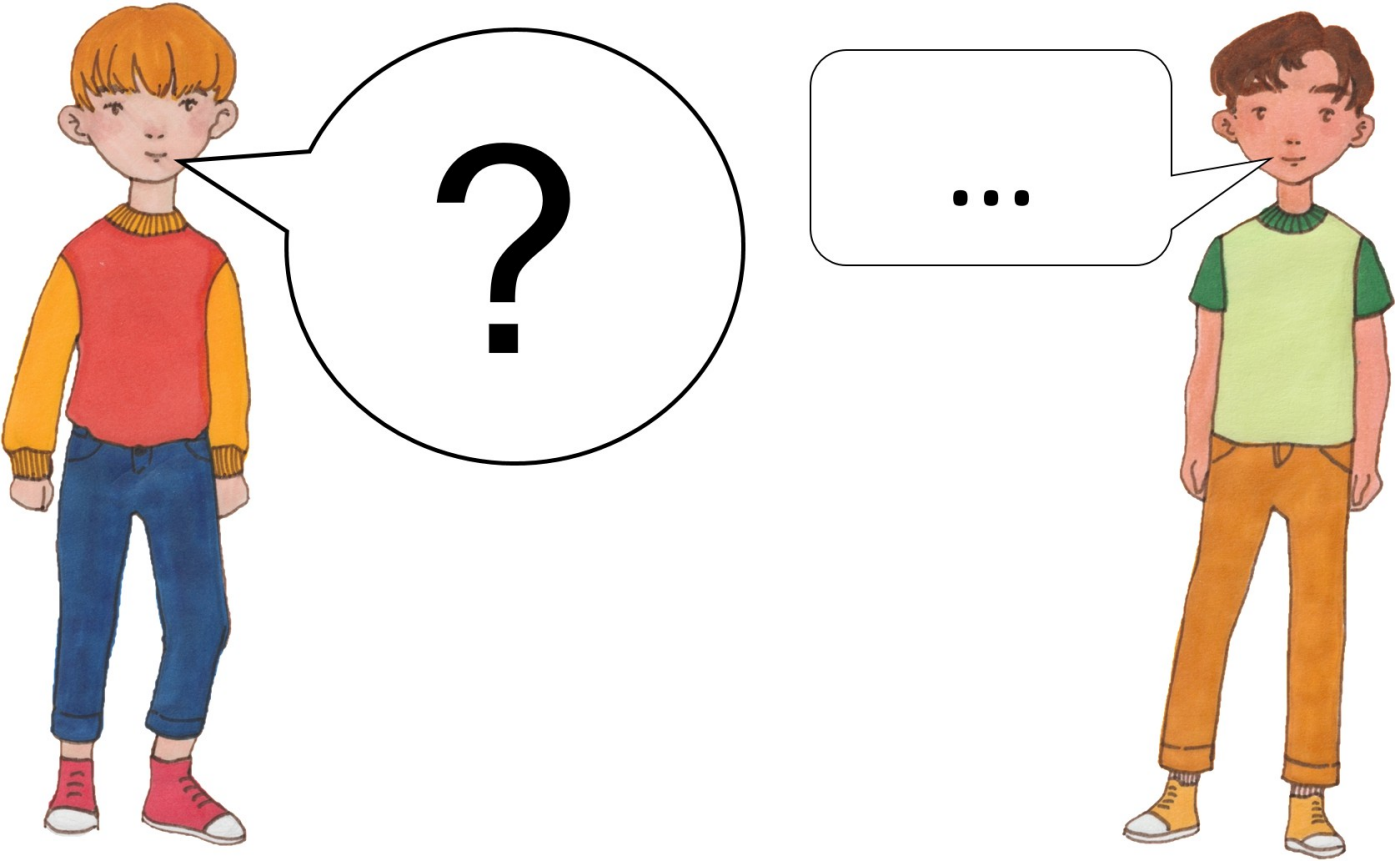
Picture of Max (left) and Tom (right) in the ingroup condition for a male participant.

As displayed in Fig 1, the experimenter finally presented a picture of both boys and told the participant that Max (who had previously donated five cookies) would ask the other boy Tom (who had previously donated one cookie) how many cookies Tom had donated. The experimenter invited the participating child to help finish the story by asking: “What do you think Tom will respond? Will he say that he donated one cookie or will he say that he donated more than one cookie?” If the participating child replied that Tom would respond “more than one”, the experimenter asked, “How many cookies will he say that he donated exactly?”, and the participant was requested to justify his response (“Why will he say that?”). Finally, the experimenter asked whether the participant thinks that Max still wants to play with Tom (collaboration question:“If Tom really says that, do you think Max still wants to play with him?”).

In the outgroup condition the procedure was the same as in the ingroup condition, except that the experimenter presented two children of the opposite gender (a boy and a girl) belonging to different groups (“tree group”, “sheep group”) and doing different things instead of collaborating on a joint activity. Parallel to the ingroup condition, the child distributing the cookies selfishly was gender-matched to the participant.

#### Additional questions

After the last story, the experimenter asked two explorative questions. First, he asked if the participant thinks that the protagonist would keep a secret for himself, if he had promised to do so (trust question). Next, the experimenter asked whether the participant wanted to be friends with the protagonist (friend question), who only donated one cookie, and why or why not he wanted to be friends with him (”Would you like to be friends with Tom? Why/why not”).

Finally, the participant was asked a question about his normative expectation: “If someone receives ten cookies and can donate some of them to another child of his group, how many cookies should one donate?”

### Coding and reliability

Children’s explanations for their predictions were classified using a modified coding scheme from Banerjee (2002) to include a moral standard category [10]. We identified whether or not the child justified their prediction(s) with reference to others’ beliefs, to situational outcomes or to moral standards by categorizing children’s answers into four categories:

Belief: Implicit or explicit reference to others’ beliefs (e.g., “otherwise Max thinks that Tom is selfish”; “So Max thinks Tom is fair”)

Outcome: Implicit or explicit reference to situational consequences of action (e.g., “So they’ll be friends”; “So Max won’t get upset”)

Moral standards: Implicit or explicit reference to social or moral standards (e.g., “One should always tell the truth”; “He should not lie”; “Because this would be fair”)

Residual: Several categories of other responses, including “Don’t know”, nonsense justifications or motives that did not clearly involve a reputational concern.

In line with Banerjee (2002) responses were initially coded as falling into one of the four categories. Theoretically, complex answers contributing to more than one category (e.g. moral standard and belief) could be coded as falling into all matching categories. However, there were no such complex answers in the current study. We derived a belief score ranging from 0 to 2 for the two test stories. Children received one point for each explanation coded as belief. Similarly, we coded a moral standards score, an outcome score and a residual score. A naïve rater, trained by the first author, coded children’s responses during the experiment. Another independent rater, trained by the first author, recoded 25% of the videos for interrater reliability. Interrater reliability was excellent with Cohen’s *κ* = 1.

### Analytic strategy

Main dependent variables were children’s predictions of the protagonist’s answer about the number of cookies and children’s normative expectations about how many cookies should be donated. Children’s justifications for their predictions were classified into four categories, as described above: Belief, Outcome, Moral standards, and Residuals. To answer the main research question whether children would predict that a person would lie for reputational concerns, we compared children’s mean predicted answer to the actual donation of one cookie with a one-sample t-test. To test whether children would predict more reputational lying towards ingroup or outgroup members, we compared their predicted answer between the ingroup and outgroup condition with a paired t-test. To check whether children expected reputational lies to approximate a normative standard, we compared children’s predicted answer to their normative expectation using a paired t-test and conducted correlation analyses between their normative expectation and predicted answer To further explain children’s predictions, we compared their justifications for predicting reputational lies and for predicting honest responses using two-sample t-tests. To examine relations between reputational lies, integrity-based trust and the willingness to be friends, we compared children who predicted lies to children who predicted honesty in their responses to the trust question and the friend question using phi coefficients. Finally, to check for differences in reputational lying between 5- and 7-year-olds, we conducted a 2 (age: 5-year-olds, 7-year-olds) × 2 (condition: ingroup, outgroup) analysis of variance (ANOVA) for children’s predicted answer. We conducted a direct age comparison for children’s normative expectations using a two-sample t-test.

## Results

### 7-year-olds

**Fig 2:**
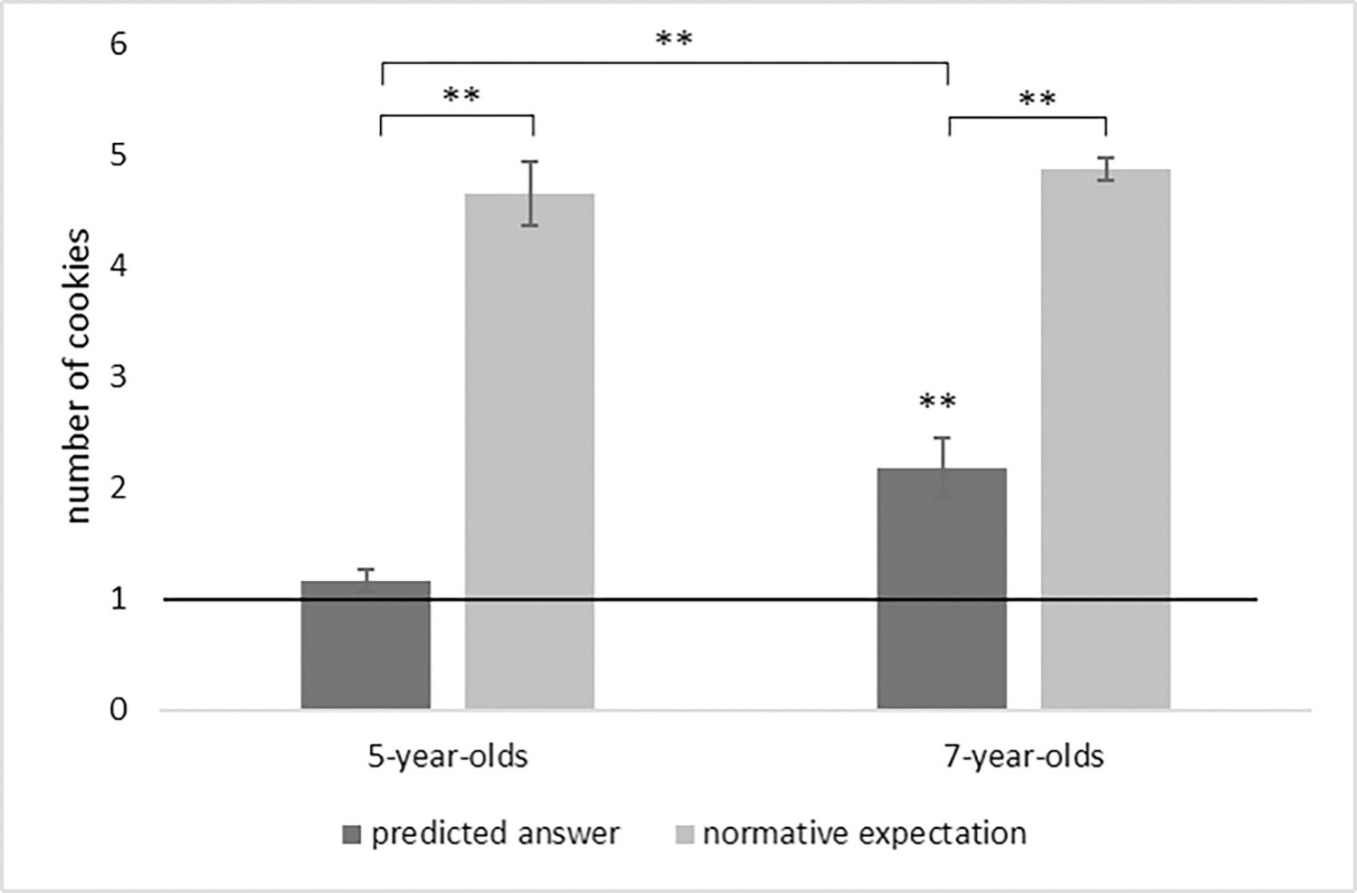
Mean predicted answer and normative expectations. Bars indicate standard errors of the mean. Stars indicate a significant difference from the real donation of one cookie or from indicated bar, **p < .001.

All children answered the control questions correctly. Forty-seven percent of children predicted that the protagonist would tell a reputational lie by stating that he had donated more than one cookie. There was a significant difference between children’s mean predicted number of donated cookies (M = 2.19, SD = 1.52) and the donation of one cookie, *t*(31) = 4.43, *p* < .001, see Fig 2. There was no difference between the predicted answer in the ingroup condition and the outgroup condition, *t*(31) = .2, *p* = .847. Children’s normative expectation (M = 4.88, SD = .55) was greater than their mean predicted answer, *t*(31) = 10.02, *p* < .001, even when children who had predicted an honest response were excluded, *t*(14) = 4.73, *p* < .001, see Fig 2. There was no significant correlation between children’s normative expectation and their predicted answer, *r*(32) = .18, *p* = .318.

Forty-one percent of reputational lies, and no prediction of honest responses, were justified with reference to how the protagonist would look to others (Belief), *t*(14) = 3.15, *p* = .007. Further, 32% of reputational lies and 5% of honest responses were justified with reference to situational consequences (Outcome), *t*(18) = 2.7, *p* = .015. No reputational lie was justified with reference to moral standards versus 17% of predictions of honest responses, *t*(25) = 1.04, *p* = .307. Twenty-seven percent of reputational lies and 74% of honest responses were justified with residual justifications, *t*(28) = 7.23, *p* < .001.

In the collaboration question, children stated more often that the two story characters wanted to play with each other, when children had predicted the protagonist to tell a reputational lie (*φ*(29) = .37, *p* = .046). Further, children more often expected the protagonist to keep a secret, when they had predicted that he would tell the truth (*φ*(30) = -.53, *p* = .004). In the friend question, children stated that they wanted to be friends with the protagonist independent of whether they predicted a reputational lie or not (*φ*(28) = -.13, *p* = .483).

### 5-year-olds

All children answered the control questions correctly. Only 3 children (9%) predicted the protagonist to tell a reputational lie by stating that he had donated more than one cookie. There was no significant difference between children’s mean predicted answer (M = 1.17, SD = .55) and the donation of one cookie, *t*(31) = 1.78, *p =* .086. There was no difference between the predicted answer in the ingroup condition and the outgroup condition, (*t*(31) = 1.23, *p* = .229). Children’s normative expectation (M = 4.66, SD = 1.64) was greater than their mean predicted answer, *t*(31) = 12.78, *p* < .001. There was no significant correlation between children’s normative expectation and their predicted answer, *r*(32) = .337, *p* = .059. Irrespectively of what children predicted the protagonist to answer, no child justified their choice with reference to how the protagonist would look to others (Belief). Fifty percent of reputational lies were justified with reference to situational outcomes. Twenty-five percent were justified with reference to moral standards and another 25% were justified with residual responses. Predictions of honest responses were never justified with reference to the situational outcomes. Ninety-five percent of predictions of honest responses were justified with residual responses and 5% were justified with reference to moral standards. Since only 3 out of 32 children predicted a reputational lie, justifications for reputational lies and honest responses were not statistically analyzed in more depth and relations with the trust question were not calculated.

### Age comparison

A 2 (age: 5-year-olds, 7-year-olds) × 2 (condition: ingroup, outgroup) analysis of variance (ANOVA) for children’s predicted answer revealed no significant effect of condition (*F*(1,62) = .18, *p* = .671, *η*_*p*_*^2^* = 003) and no significant interaction of condition and age (*F*(1,62) = .59, *p* = .446, *η*_*p*_*^2^* = 009). However, results revealed a significant effect of age (*F*(1, 62) = 12.68, *p* = .001, *η*_*p*_*^2^* = .17). As displayed in Fig 2, the mean predicted number of donated cookies was significantly higher in 7-year-olds than in 5-year-olds (*t*(39) = 3.56, *p* < .001). More 7- than 5-year-olds predicted a reputational lie, Fisher’s exact test *p* = .002. Children’s normative expectations were not different between the two age groups, (*t*(38) = .72, *p* = .479).

## Discussion

The current study tested children’s explicit understanding of reputational lying in a third person in the context of prosocial behavior. Seven-year-olds as a group predicted that a protagonist who had distributed a resource selfishly would tell a reputational lie by stating that he had donated more than he actually did. The predicted answer entailed higher amounts of donation in 7- than in 5-year-olds; in fact, neglectable few 5-year-olds predicted an answer that would deviate from the truth at all. This suggests that with age children become increasingly able to understand how reputational motives shape others’ behaviors and lead to lying. Seven-year-olds frequently justified their prediction of a reputational lie with reference to how the protagonist would look to others. The finding reveals that around the age of 7 years, children begin to develop an explicit conceptual understanding of a reputational mind. In contrast, findings from the current study suggest that 5-year-olds still struggle with predicting reputational lying in third party interactions, despite their skills at telling reputational lies themselves in direct interactions with peers [6]. Further, their justifications for their predictions never entailed reference to others’ beliefs about the protagonist.

This is in contrast to results showing that in some contexts, children as young as 5 years of age predict that someone with reputational motives will avoid behavior that might harm her reputation (i.e., seek help publicly after failure) [15]. However, this latter study made use of forced-choice questions and it remains unclear whether children would have taken the characters’ reputational concerns into account, if they had been asked to justify the behavior themselves. In the current study, it is theoretically possible that additional procedural demands masked 5-year-olds competence. However, all children answered the control questions correctly, suggesting that 5-year-olds did not have difficulties with understanding the general procedure or stories. Furthermore, 5-year-olds clearly did have a normative expectation about how many cookies a person should share. It might be possible that 5-year-olds did not predict reputational lying because they judged lying morally wrong in any circumstances. However, only 5% of predictions of honest responses were justified with reference to moral standards. It thus seems unlikely that 5-year-olds refrain from predicting reputational lying because of their moral condemnation of lie-telling in general. Furthermore, they do lie themselves in a similar situation [6]. The lack of competence in predicting others’ reputational lying towards third persons is in contrast to their own reputational lying in direct peer interactions [6]. Likely, it reflects a developmental difference from a practical to a more conceptual, theory-like understanding of reputation management. We suggest that while a practical understanding of reputational lying surfaces in second-person engagement around the age of 5 years, a conceptual, theory-like understanding extending to predictions and justifications of others’ behaviors in hypothetical third-party interactions begins to emerge around the age of 7 years.

Results did not reveal differences between predicting reputational lying towards ingroup and outgroup members in neither age group. This is in contrast to research showing that in social interactions with others, children as young as 5 years exhibit a group bias and care more about their reputation with ingroup members and potential reciprocators [19]. One possibility is that this in/out-group effect does not apply to the particular strategy of reputational lies, as group biases in this context are still poorly understood [6]. Furthermore, the stories used in the current study did not include a competitive group setting. It is possible that these stories did not emphasize the different group belongings to a level that it was relevant to the participant. Possibly, a stronger manipulation (with higher stakes in the character’s competition or cooperation) would result in a group bias in line with previous research [19, 20], that is, in more reputational lying towards an ingroup than towards an outgroup member.

Children’s normative expectation of how many cookies one should share with another child was greater than what they predicted the protagonist to answer, also when excluding those children who expected no lie. This suggests that children did not necessarily expect reputational lies to approximate the normative expectation (i.e., a fair distribution). One possibility could be that children are aware that trying to promote one’s own reputation by making too positive claims about oneself can have negative reputational consequences [26, 27]. It is also possible that children realistically predicted that others do not always, or fully, behave according to social norms, and so they adapted their predicted answer accordingly, i.e. they factored in the individual’s tendencies as well. Another possibility is that children balance the severity of a lie with the potential reputational benefit. Even 7-year-olds likely place weight on being honest, and so a more severe lie (e.g., saying that you gave more than would be expected realistically or even normatively) might outweigh the potential reputational benefit. This could be due to the belief that either the lie would seem unbelievable, or the belief that even the selfish distributor would be uncomfortable telling such a severe lie.

Findings of the current study reveal that beginning around 7 years, children are not only able to reason about their own reputation explicitly, e.g. when they are asked to imagine themselves as the protagonist and to judge how they would feel and what they would do [13], but that they also increasingly predict self-promoting strategies such as reputational lying when asked about a third person. Furthermore, they justify their prediction of reputational lies with reference to how the protagonist would look to others and with reference to situational outcomes, e.g. to avoid losing a friend. In contrast to younger children, 7-year-olds begin to explicitly understand lying as reputational strategy including justifying their predictions and anticipating further consequences.

Seven- year-olds stated that the two story characters wanted to play with each other, if the protagonist had told a reputational lie and, in consequence, had appeared fair to the other character. They thus were able to understand that reputational lying might prevent someone who had previously behaved selfishly from being excluded of a group. In addition, the trust question revealed that 7-year-olds expect children who tell the truth to keep a secret, that is to be loyal to their group. Relative to honesty, reputational lies seem to harm trust and the willingness to rely on the other person’s word or promise. Children who predicted a reputational lie evaluated the protagonist as less trustworthy and expected him to keep a promise less often than children who predicted honesty. This is in line with prior findings on the relation between prosocial lies and trustworthiness with adults [21]. Reputational lies did not clearly affect the willingness to play with each other, that is the willingness to collaborate. This might be explained by the fact that trust and commitment are key aspects of human collaboration [1]. Reputational lies, on the one hand, might question a person’s truthfulness, while on the other hand, they signal an adaptability and commitment to the group. Further research is needed to examine the relation between reputational lies and the willingness to cooperate in more depth.

## Supporting information

S1 File Supplementary material. (DOCX)
