# Supplementary material for: Being honest won’t pay. Seven- but not 5-year-olds begin to predict that others will lie for reputational reasons
Source: PLoS One. 2025 Jan 10;20(1):e0317334. doi: 10.1371/journal.pone.0317334 (PMC11723524; doi:10.1371/journal.pone.0317334)
Supplement: S1 File — (DOCX) [file pone.0317334.s001.docx]

# S1. Supporting Information

## Practice Story

*E presents a picture of a lion.* Do you see the lion? (…)

*E presents a picture of a chocolate*. This chocolate belongs to the lion. Do you see the chocolate? (…)

*E presents a picture of a duck.* This is the duck.

Now I will ask you a question. To whom does this chocolate belong? (…)

## Test story – ingroup condition (male participant)

*E presents a picture of two boys.* This are Tom and Max.

*E presents a star symbol.* They belong to the star group. In the star group, they build a tree house together.

*E presents a picture of Tom*. When Tom is alone, he receives ten cookies. He really likes cookies. Do you like cookies? (…) Tom can decide whether he wants to keep all cookies to himself or donate some of the cookies to another child of his group. Tom thinks ‘I will give one cookie away and keep the rest to myself, because I want to eat nine cookies.’ Tom keeps nine cookies to himself and donates one cookies to another child of his group.

Did you listen carefully? I now have some questions for you.

To what group does Tom belong? (…) *Child is corrected, if answer is wrong*.

How many cookies did Tom donate to the other child? (…) *If correct answer:* Yes, he gave one cookie to the other child and kept nine cookies to himself. *If wrong answer*: How many cookies did Tom keep to himself? (…) Yes, he gave one cookie to the other child and kept nine cookies to himself.

*E presents a picture of Max*. This is Max. Max belongs to the same group as Tom, the star group. When Max is alone, he also receives ten cookies. Max can decide whether he wants to keep all cookies to himself or donate some of the cookies to another child of his group. Max thinks ‘I will give five cookies away and keep five cookies to myself, because I consider this fair. I’ll eat my cookies later.’ Max keeps five cookies to himself and donates five cookies to another child of his group.

Did you listen carefully? I now have some questions for you.

To what group does Max belong? (…) *Child is corrected, if answer is wrong.*

How many cookies did Max donate to the other child? (…) *If correct answer:* Yes, he gave five cookies to the other child and kept five cookies to himself. *If wrong answer*: How many cookies did Max keep to himself? (…) Yes, he gave five cookies to the other child and kept five cookies to himself.

*E presents a picture of Max and Tom.* Look, here are Max and Tom again. Max is curious. He asks: “Tom, how many cookies did you give to the other child?”

Now, it’s your turn, *name of the participant*. You can continue the story. What do you think Tom will respond? Will he say that he donated one cookie or will he say that he donated more than one cookie? (…) *If ‘more than one cookie’*: How many? (…) Why will he say that?

What do you think - does Max still want to play with Tom? (…)

## Test story - outgroup condition (male participant)

*E presents a picture of a boy and a girl.* This is Tom and this is Clara.

*E presents a star symbol and a tree symbol.* They belong to different groups and do different things. Tom belongs to the tree group and Clara belongs to the star group.

*E presents a picture of Tom*. When Tom is alone, he receives ten cookies. He really likes cookies. Do you like cookies? (…) Tom can decide whether he wants to keep all cookies to himself or donate some of the cookies to another child of his group. Tom thinks ‘I will give one cookie away and keep the rest to myself, because I want to eat nine cookies.’ Tom keeps nine cookies to himself and donates one cookies to another child of his group.

Did you listen carefully? I how have some questions for you.

To what group does Tom belong? (…) *Child is corrected, if answer is wrong*.

How many cookies did Tom donate to the other child? (…) *If correct answer:* Yes, he gave one cookie to the other child and kept nine cookies to himself. *If wrong answer*: How many cookies did Tom keep to himself? (…) Yes, he gave one cookie to the other child and kept nine cookies to himself.

*E presents a picture of Clara*. This is Clara. Clara does not belong to the tree group, but she belongs to the star group. When Clara is alone, she also receives ten cookies. Clara can decide whether she wants to keep all cookies to herself or donate some of the cookies to another child of her group. Clara thinks ‘I will give five cookies away and keep five cookies to myself, because I consider this fair. I’ll eat my cookies later.’ Clara keeps five cookies to herself and donates five cookies to another child of her group.

Did you listen carefully? I now have some questions for you.

To what group does Clara belong? (…) *Child is corrected, if answer is wrong.*

How many cookies did Clara donate to the other child? (…) *If correct answer:* Yes, she gave five cookies to the other child and kept five cookies to herself. *If wrong answer*: How many cookies did Clara keep to herself? (…) Yes, she gave five cookies to the other child and kept five cookies to herself.

*E presents a picture of Clara and Tom.* Look, here are Clara and Tom again. Clara is curious. She asks: “Tom, how many cookies did you give to the other child?”

Now, it’s your turn, *name of the participant*. You can continue the story. What do you think Tom will respond? Will he say that he donated one cookie or will he say that he donated more than one cookie? (…) *If ‘more than one cookie’*: How many? (…) Why will he say that?

What do you think - does Clara still want to play with Tom? (…)

## Additional questions

*Trust question*: If Tom promised to keep a secret, do you think he would keep that promise? (…)

*Friend question:* Would you like to be friends with Tom? (…) Why/Why not? (…)

*Normative expectation*: If someone receives ten cookies and can donate some of them to another child of his group, how many cookies should one donate? (…)
